# *N*-(2,6-Dichloro­phen­yl)-4-methyl­benzene­sulfonamide

**DOI:** 10.1107/S1600536810051792

**Published:** 2010-12-15

**Authors:** K. Shakuntala, Sabine Foro, B. Thimme Gowda

**Affiliations:** a Department of Chemistry, Mangalore University, Mangalagangotri 574 199, Mangalore, India; b Institute of Materials Science, Darmstadt University of Technology, Petersenstrasse 23, D-64287 Darmstadt, Germany

## Abstract

In the title compound, C_13_H_11_Cl_2_NO_2_S, the mol­ecule is bent at the S atom with a C—SO_2_—NH—C torsion angle of −90.4 (2)°. The sulfonyl benzene and the aniline benzene rings are tilted relative to each other by 51.7 (1)°. In the crystal, mol­ecules are linked by N— H⋯O inter­actions into chains with graph-set notation *C*(4) along [100].

## Related literature

For our study of the effect of substituents on the structures of *N*-(ar­yl)aryl­sulfonamides, see: Gowda *et al.* (2009 ▶); Nirmala *et al.* (2010 ▶); Shakuntala *et al.* (2010 ▶). For related structures, see: Gelbrich *et al.* (2007 ▶); Perlovich *et al.* (2006 ▶). For hydrogen-bond motifs, see: Bernstein *et al.* (1995 ▶).
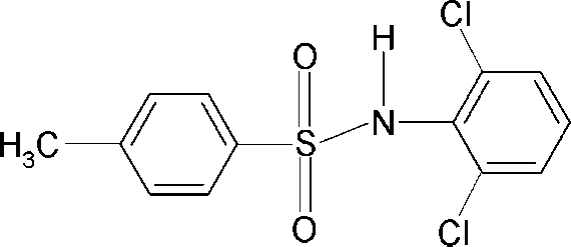


## Experimental

### 

#### Crystal data

C_13_H_11_Cl_2_NO_2_S
*M* *_r_* = 316.19
Monoclinic, 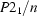

*a* = 5.0456 (6) Å
*b* = 17.128 (2) Å
*c* = 16.540 (2) Å
β = 97.13 (1)°
*V* = 1418.4 (3) Å^3^
*Z* = 4
Mo *K*α radiation
μ = 0.60 mm^−1^
*T* = 293 K
0.55 × 0.28 × 0.25 mm

#### Data collection

Oxford Diffraction Xcalibur diffractometer with a Sapphire CCD detector
Absorption correction: multi-scan (*CrysAlis RED*; Oxford Diffraction, 2009 ▶) *T*_min_ = 0.734, *T*_max_ = 0.864
5631 measured reflections
2904 independent reflections
2493 reflections with *I* > 2σ(*I*)
*R*_int_ = 0.011

#### Refinement

*R*[*F* ^2^ > 2σ(*F* ^2^)] = 0.038
*wR*(*F* ^2^) = 0.102
*S* = 1.05
2904 reflections
176 parameters
1 restraint
H atoms treated by a mixture of independent and constrained refinement
Δρ_max_ = 0.30 e Å^−3^
Δρ_min_ = −0.48 e Å^−3^

### 

Data collection: *CrysAlis CCD* (Oxford Diffraction, 2009 ▶); cell refinement: *CrysAlis RED* (Oxford Diffraction, 2009 ▶); data reduction: *CrysAlis RED*; program(s) used to solve structure: *SHELXS97* (Sheldrick, 2008 ▶); program(s) used to refine structure: *SHELXL97* (Sheldrick, 2008 ▶); molecular graphics: *PLATON* (Spek, 2009 ▶); software used to prepare material for publication: *SHELXL97*.

## Supplementary Material

Crystal structure: contains datablocks I, global. DOI: 10.1107/S1600536810051792/bx2335sup1.cif

Structure factors: contains datablocks I. DOI: 10.1107/S1600536810051792/bx2335Isup2.hkl

Additional supplementary materials:  crystallographic information; 3D view; checkCIF report

## Figures and Tables

**Table 1:** Hydrogen-bond geometry (Å, °)

*D*—H⋯*A*	*D*—H	H⋯*A*	*D*⋯*A*	*D*—H⋯*A*
N1—H1*N*⋯O1^i^	0.83 (2)	2.16 (2)	2.971 (2)	165 (2)

Symmetry code: (i) 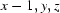.

**Fig. 1.**
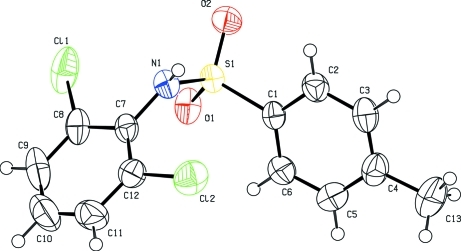
Molecular structure of (I), showing the atom labelling scheme and displacement ellipsoids are drawn at the 50% probability level.

**Fig. 2.**
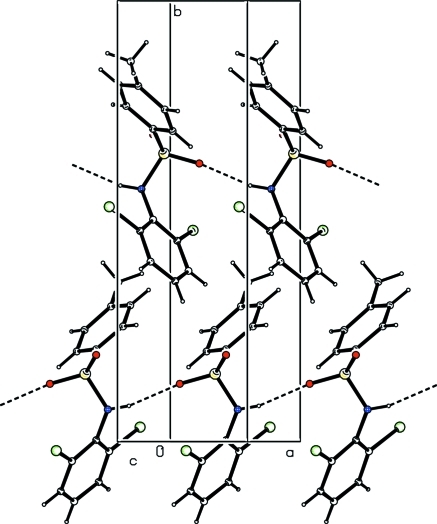
Molecular packing of (I) with hydrogen bonding shown as dashed lines.

## supplementary crystallographic information

### Comment

In the present work, as part of a study of the substituent effects on the crystal structures of *N*-(aryl)arylsulfonamides (Gowda *et al.*, 2009; Nirmala *et al.*, 2010; Shakuntala *et al.*, 2010), the structure of *N*-(2,6-dichlorophenyl)-4-methylbenzenesulfonamide (I) has been determined (Fig. 1). The conformation of the N—H bond orients away from the two *ortho*-chloro groups in the adjacent benzene ring.

The molecule in (I) is bent at the S atom with the C—SO_2_—NH—C torsion angle of -90.4 (2)°, compared to the values of 88.0 (2)° in *N*-(2,6-dimethylphenyl)-4-methylbenzenesulfonamide (II) (Nirmala *et al.*, 2010), 65.4 (2)° (molecule 1) and -61.7 (2)° (molecule 2) in *N*-(2,3-dichlorophenyl)-4-methylbenzenesulfonamide (III) (Shakuntala *et al.*, 2010) and 69.3 (4)° in *N*-(3,5-dichlorophenyl)-4-methylbenzenesulfonamide (IV) (Gowda *et al.*, 2009).

The two benzene rings in (I) are tilted relative to each other by 51.7 (1)°, compared to the values of 49.8 (1)° in (II). 76.0 (1)° (molecule 1) and 79.9 (1)° (molecule 2) in (III) and 79.6 (1)° in (IV).

The other bond parameters in (I) are similar to those observed in (II), (III), (IV) and other aryl sulfonamides (Perlovich *et al.*, 2006; Gelbrich *et al.*, 2007).

In the crystal, molecules are linked by N— H··· O interaction into chain with graph-set notation C(4) along [100] (Bernstein *et al.*, 1995), Fig. 2.

### Experimental

The solution of toluene (10 ml) in chloroform (40 ml) was treated dropwise with chlorosulfonic acid (25 ml) at 0 ° C. After the initial evolution of hydrogen chloride subsided, the reaction mixture was brought to room temperature and poured into crushed ice in a beaker. The chloroform layer was separated, washed with cold water and allowed to evaporate slowly. The residual 4-methylbenzenesulfonylchloride was treated with 2,6-dichloroaniline in the stoichiometric ratio and boiled for ten minutes. The reaction mixture was then cooled to room temperature and added to ice cold water (100 ml). The resultant *N*-(2,6-dichlorophenyl)-4-methylbenzenesulfonamide was filtered under suction and washed thoroughly with cold water. It was then recrystallized to constant melting point from dilute ethanol. The purity of the compound was checked and characterized by recording its infrared and NMR spectra.

Rod like colourless single crystals used in X-ray diffraction studies were grown in ethanolic solution by slow evaporation at room temperature.

### Refinement

The H atom of the NH group was located in a difference map and later restrained to the distance N—H = 0.86 (2) Å. The other H atoms were positioned with idealized geometry using a riding model with C—H = 0.93–0.96 Å H-atoms were refined with isotropic displacement parameters (set to 1.2 times of the *U*_eq_ of the parent atom).

### Crystal data

**Table:**

C_13_H_11_Cl_2_NO_2_S	*F*(000) = 648
*M**_r_* = 316.19	*D*_x_ = 1.481 Mg m^−^^3^
Monoclinic, *P*2_1_/*n*	Mo *K*α radiation, λ = 0.71073 Å
Hall symbol: -P 2yn	Cell parameters from 3098 reflections
*a* = 5.0456 (6) Å	θ = 3.4–27.7°
*b* = 17.128 (2) Å	µ = 0.60 mm^−^^1^
*c* = 16.540 (2) Å	*T* = 293 K
β = 97.13 (1)°	Rod, colourless
*V* = 1418.4 (3) Å^3^	0.55 × 0.28 × 0.25 mm
*Z* = 4	

### Data collection

**Table:**

Oxford Diffraction Xcalibur diffractometer with a Sapphire CCD detector	2904 independent reflections
Radiation source: fine-focus sealed tube	2493 reflections with *I* > 2σ(*I*)
graphite	*R*_int_ = 0.011
Rotation method data acquisition using ω and phi scans	θ_max_ = 26.4°, θ_min_ = 3.4°
Absorption correction: multi-scan (*CrysAlis RED*; Oxford Diffraction, 2009)	*h* = −3→6
*T*_min_ = 0.734, *T*_max_ = 0.864	*k* = −21→18
5631 measured reflections	*l* = −20→15

### Refinement

**Table:**

Refinement on *F*^2^	Primary atom site location: structure-invariant direct methods
Least-squares matrix: full	Secondary atom site location: difference Fourier map
*R*[*F*^2^ > 2σ(*F*^2^)] = 0.038	Hydrogen site location: inferred from neighbouring sites
*wR*(*F*^2^) = 0.102	H atoms treated by a mixture of independent and constrained refinement
*S* = 1.05	*w* = 1/[σ^2^(*F*_o_^2^) + (0.0469*P*)^2^ + 0.7136*P*] where *P* = (*F*_o_^2^ + 2*F*_c_^2^)/3
2904 reflections	(Δ/σ)_max_ = 0.028
176 parameters	Δρ_max_ = 0.30 e Å^−^^3^
1 restraint	Δρ_min_ = −0.48 e Å^−^^3^

### Special details

**Table:**

Experimental. CrysAlis RED (Oxford Diffraction, 2009) Empirical absorption correction using spherical harmonics, implemented in SCALE3 ABSPACK scaling algorithm.
Geometry. All e.s.d.'s (except the e.s.d. in the dihedral angle between two l.s. planes) are estimated using the full covariance matrix. The cell e.s.d.'s are taken into account individually in the estimation of e.s.d.'s in distances, angles and torsion angles; correlations between e.s.d.'s in cell parameters are only used when they are defined by crystal symmetry. An approximate (isotropic) treatment of cell e.s.d.'s is used for estimating e.s.d.'s involving l.s. planes.
Refinement. Refinement of *F*^2^ against ALL reflections. The weighted *R*-factor *wR* and goodness of fit *S* are based on *F*^2^, conventional *R*-factors *R* are based on *F*, with *F* set to zero for negative *F*^2^. The threshold expression of *F*^2^ > σ(*F*^2^) is used only for calculating *R*-factors(gt) *etc*. and is not relevant to the choice of reflections for refinement. *R*-factors based on *F*^2^ are statistically about twice as large as those based on *F*, and *R*- factors based on ALL data will be even larger.

### Fractional atomic coordinates and isotropic or equivalent isotropic displacement parameters (Å^2^)

**Table:**

	*x*	*y*	*z*	*U*_iso_*/*U*_eq_	
C1	0.0019 (4)	0.70942 (11)	0.33030 (12)	0.0361 (4)	
C2	−0.2076 (4)	0.76146 (13)	0.32461 (14)	0.0471 (5)	
H2	−0.3280	0.7645	0.2773	0.057*	
C3	−0.2359 (5)	0.80913 (14)	0.39037 (16)	0.0559 (6)	
H3	−0.3784	0.8439	0.3869	0.067*	
C4	−0.0594 (5)	0.80678 (14)	0.46089 (15)	0.0537 (6)	
C5	0.1467 (5)	0.75322 (15)	0.46540 (15)	0.0582 (6)	
H5	0.2656	0.7498	0.5130	0.070*	
C6	0.1799 (5)	0.70457 (14)	0.40068 (14)	0.0508 (5)	
H6	0.3202	0.6690	0.4045	0.061*	
C7	−0.0179 (4)	0.50337 (11)	0.28274 (13)	0.0387 (4)	
C8	0.1533 (5)	0.45552 (13)	0.24465 (16)	0.0521 (6)	
C9	0.2543 (6)	0.38721 (15)	0.2808 (2)	0.0736 (8)	
H9	0.3728	0.3570	0.2554	0.088*	
C10	0.1807 (7)	0.36409 (16)	0.3536 (2)	0.0793 (9)	
H10	0.2488	0.3180	0.3776	0.095*	
C11	0.0071 (6)	0.40828 (15)	0.39182 (17)	0.0674 (7)	
H11	−0.0466	0.3917	0.4408	0.081*	
C12	−0.0877 (4)	0.47785 (13)	0.35667 (14)	0.0471 (5)	
C13	−0.0895 (7)	0.8623 (2)	0.5296 (2)	0.0855 (9)	
H13A	−0.2690	0.8819	0.5242	0.103*	
H13B	0.0328	0.9050	0.5278	0.103*	
H13C	−0.0513	0.8354	0.5807	0.103*	
N1	−0.1228 (3)	0.57274 (10)	0.24474 (10)	0.0388 (4)	
H1N	−0.286 (3)	0.5809 (14)	0.2422 (14)	0.047*	
O1	0.3260 (3)	0.63094 (9)	0.25207 (10)	0.0495 (4)	
O2	−0.0611 (3)	0.69646 (9)	0.17450 (9)	0.0538 (4)	
Cl1	0.23560 (17)	0.47929 (4)	0.14965 (5)	0.0797 (3)	
Cl2	−0.30503 (14)	0.53278 (4)	0.40686 (4)	0.0675 (2)	
S1	0.05144 (9)	0.65342 (3)	0.24407 (3)	0.03684 (15)	

### Atomic displacement parameters (Å^2^)

**Table:**

	*U*^11^	*U*^22^	*U*^33^	*U*^12^	*U*^13^	*U*^23^
C1	0.0349 (9)	0.0306 (9)	0.0445 (10)	−0.0027 (7)	0.0116 (8)	−0.0009 (8)
C2	0.0412 (11)	0.0452 (12)	0.0547 (12)	0.0063 (9)	0.0046 (9)	−0.0015 (10)
C3	0.0526 (13)	0.0481 (13)	0.0695 (15)	0.0120 (10)	0.0170 (12)	−0.0079 (11)
C4	0.0646 (14)	0.0481 (12)	0.0520 (13)	−0.0031 (11)	0.0211 (11)	−0.0068 (10)
C5	0.0648 (15)	0.0631 (15)	0.0456 (12)	0.0016 (12)	0.0021 (10)	−0.0030 (11)
C6	0.0508 (12)	0.0485 (12)	0.0526 (12)	0.0097 (10)	0.0045 (10)	0.0011 (10)
C7	0.0349 (10)	0.0309 (9)	0.0502 (11)	−0.0052 (8)	0.0040 (8)	−0.0045 (8)
C8	0.0499 (12)	0.0367 (11)	0.0716 (15)	−0.0060 (9)	0.0150 (11)	−0.0127 (10)
C9	0.0655 (16)	0.0384 (13)	0.117 (3)	0.0088 (12)	0.0133 (16)	−0.0165 (15)
C10	0.085 (2)	0.0417 (14)	0.105 (3)	0.0090 (14)	−0.0109 (18)	0.0073 (15)
C11	0.0817 (18)	0.0513 (14)	0.0654 (16)	−0.0093 (13)	−0.0055 (13)	0.0132 (12)
C12	0.0463 (12)	0.0438 (11)	0.0502 (12)	−0.0056 (9)	0.0021 (9)	−0.0019 (9)
C13	0.104 (2)	0.086 (2)	0.0706 (18)	0.0060 (19)	0.0242 (17)	−0.0281 (17)
N1	0.0267 (7)	0.0374 (9)	0.0524 (10)	−0.0016 (7)	0.0047 (7)	−0.0004 (7)
O1	0.0310 (7)	0.0471 (8)	0.0728 (10)	−0.0049 (6)	0.0158 (7)	−0.0143 (8)
O2	0.0686 (10)	0.0477 (9)	0.0462 (8)	0.0004 (8)	0.0111 (7)	0.0086 (7)
Cl1	0.1007 (6)	0.0582 (4)	0.0912 (5)	−0.0164 (4)	0.0558 (4)	−0.0252 (3)
Cl2	0.0720 (4)	0.0775 (4)	0.0574 (4)	0.0006 (3)	0.0258 (3)	−0.0011 (3)
S1	0.0334 (2)	0.0338 (3)	0.0449 (3)	−0.00172 (19)	0.01091 (19)	−0.00076 (19)

### Geometric parameters (Å, °)

**Table:**

C1—C2	1.377 (3)	C8—Cl1	1.723 (3)
C1—C6	1.382 (3)	C9—C10	1.363 (4)
C1—S1	1.7620 (19)	C9—H9	0.9300
C2—C3	1.381 (3)	C10—C11	1.370 (4)
C2—H2	0.9300	C10—H10	0.9300
C3—C4	1.377 (4)	C11—C12	1.385 (3)
C3—H3	0.9300	C11—H11	0.9300
C4—C5	1.382 (3)	C12—Cl2	1.733 (2)
C4—C13	1.504 (3)	C13—H13A	0.9600
C5—C6	1.383 (3)	C13—H13B	0.9600
C5—H5	0.9300	C13—H13C	0.9600
C6—H6	0.9300	N1—S1	1.6386 (17)
C7—C12	1.385 (3)	N1—H1N	0.830 (16)
C7—C8	1.396 (3)	O1—S1	1.4283 (14)
C7—N1	1.416 (3)	O2—S1	1.4241 (16)
C8—C9	1.382 (4)		
			
C2—C1—C6	120.64 (19)	C8—C9—H9	119.9
C2—C1—S1	118.80 (16)	C9—C10—C11	120.5 (3)
C6—C1—S1	120.44 (15)	C9—C10—H10	119.8
C1—C2—C3	118.8 (2)	C11—C10—H10	119.8
C1—C2—H2	120.6	C10—C11—C12	119.3 (3)
C3—C2—H2	120.6	C10—C11—H11	120.4
C4—C3—C2	122.0 (2)	C12—C11—H11	120.4
C4—C3—H3	119.0	C7—C12—C11	122.0 (2)
C2—C3—H3	119.0	C7—C12—Cl2	119.81 (17)
C3—C4—C5	117.9 (2)	C11—C12—Cl2	118.2 (2)
C3—C4—C13	120.4 (2)	C4—C13—H13A	109.5
C5—C4—C13	121.6 (3)	C4—C13—H13B	109.5
C4—C5—C6	121.4 (2)	H13A—C13—H13B	109.5
C4—C5—H5	119.3	C4—C13—H13C	109.5
C6—C5—H5	119.3	H13A—C13—H13C	109.5
C1—C6—C5	119.2 (2)	H13B—C13—H13C	109.5
C1—C6—H6	120.4	C7—N1—S1	122.66 (13)
C5—C6—H6	120.4	C7—N1—H1N	118.4 (17)
C12—C7—C8	116.9 (2)	S1—N1—H1N	112.8 (17)
C12—C7—N1	122.35 (18)	O2—S1—O1	119.96 (10)
C8—C7—N1	120.7 (2)	O2—S1—N1	106.35 (9)
C9—C8—C7	121.2 (3)	O1—S1—N1	106.68 (9)
C9—C8—Cl1	118.5 (2)	O2—S1—C1	106.85 (10)
C7—C8—Cl1	120.36 (19)	O1—S1—C1	107.77 (9)
C10—C9—C8	120.1 (3)	N1—S1—C1	108.88 (9)
C10—C9—H9	119.9		
			
C6—C1—C2—C3	0.4 (3)	C8—C7—C12—C11	0.1 (3)
S1—C1—C2—C3	−175.68 (18)	N1—C7—C12—C11	177.3 (2)
C1—C2—C3—C4	0.8 (4)	C8—C7—C12—Cl2	−178.62 (16)
C2—C3—C4—C5	−1.7 (4)	N1—C7—C12—Cl2	−1.4 (3)
C2—C3—C4—C13	177.1 (3)	C10—C11—C12—C7	1.7 (4)
C3—C4—C5—C6	1.5 (4)	C10—C11—C12—Cl2	−179.5 (2)
C13—C4—C5—C6	−177.2 (3)	C12—C7—N1—S1	104.6 (2)
C2—C1—C6—C5	−0.6 (3)	C8—C7—N1—S1	−78.4 (2)
S1—C1—C6—C5	175.45 (18)	C7—N1—S1—O2	154.79 (16)
C4—C5—C6—C1	−0.4 (4)	C7—N1—S1—O1	25.65 (18)
C12—C7—C8—C9	−2.0 (3)	C7—N1—S1—C1	−90.40 (17)
N1—C7—C8—C9	−179.2 (2)	C2—C1—S1—O2	25.70 (19)
C12—C7—C8—Cl1	176.09 (16)	C6—C1—S1—O2	−150.40 (17)
N1—C7—C8—Cl1	−1.1 (3)	C2—C1—S1—O1	155.87 (16)
C7—C8—C9—C10	2.1 (4)	C6—C1—S1—O1	−20.2 (2)
Cl1—C8—C9—C10	−176.0 (2)	C2—C1—S1—N1	−88.79 (17)
C8—C9—C10—C11	−0.2 (5)	C6—C1—S1—N1	95.11 (18)
C9—C10—C11—C12	−1.7 (4)		

### Hydrogen-bond geometry (Å, °)

**Table:**

*D*—H···*A*	*D*—H	H···*A*	*D*···*A*	*D*—H···*A*
N1—H1N···O1^i^	0.83 (2)	2.16 (2)	2.971 (2)	165 (2)

Symmetry codes: (i) *x*−1, *y*, *z*.
